# Report of the Diseases of Edinburgh for May, 1810

**Published:** 1810-07

**Authors:** John Roberton


### *Report of the Diseases of Edinburgh for May,* 1810.

By John Roberton, M. D.

This is in general, in this part of the world, a cold and disagreeable month. On the present occasion it has not deviated from what it usually is. It commenced with very cold easterly winds and some showers of rain, and the city was enveloped in fogs of considerable density, rendering the weather extremely uncomfortable; this was succeeded by partial showers of hail and snow, which, during most part of two days, fell in great abundance. The neighbouring hills were entirely covered by the snow, and, in every respect, the weather had more the appearance of January than May. This was succeeded by days of heavy rain, and this again by firm frost, which produced ice of considerable thickness. We had then a fall of hail and snow, and the wind became very boisterous. Indeed, with the exception of one or two days about the end of the month, and now and then a part of one day or so throughout the whole of it, the weather was very bad.

The barometer was in general low throughout the whole month.

The thermometer varied considerably, but was also in general rather low, sometimes indeed below the freezing point; and a part of one or two days it was as high as 60°.

The diseases have been for the most part either of a slightly inflammatory sort, or consequences of such a state of the system, but few of them, seem very severe; and, independently of the unfavourable state of the weather, the general health of the people seems to be improving.

Chin-cough seems still to exist amoug us, and in various places it has even increased both in extent and severity. The inflammatory state of the system mentioned last month, seems not so evidently marked as formerly, but consequences of such a state seem pretty frequently to be met with in practice; such as abscesses, ulcers, and effusions of serum in different parts of the body. Stomach complaints, and diarrhœa, seem to have abated in their violence as well as in their frequency of occurrence in practice. The other complaints, except catarrh and cynanche tonsillaris, seem also to have abated. These, however, probably from the damp and cold weather, are still to be met with of very considerable severity.

The prevailing complaints then of the present month seem to be chin-cough, catarrh, cynanche tonsillaris, abscesses, ulcers, and dropsical effusions.

Of

Of the first three of these complaints I have previously said enough, both with regard to their nature and treatment.

With regard to the abscesses and ulcers, they have appeared rather more plentiful than I recollect to have seen them in practice. Although there can be little doubt that the existence of these affections is owing, in general, to the previous inflammatory state of the system, yet, from the comparative mildness of that inflammatory state during the winter, probably there may be some other cause of the nature and particular influence of which we are not so well acquainted. For if it were alone owing to such an inflammatory diathesis, the present state of such ulcers, &c. would be milder than usual, rather than, as is evident, their appearing in an aggravated degree. Other seasons too, when the inflammatory state of the system prevailed in a very great degree, were not so certainly followed by such ulcers, &c. as on the present occasion, when, from that circumstance, we had reason to expect the appearance of such affections. This is one among the many reasons which obtrude themselves upon us, and which show the necessity of particular rather than general reasoning respecting these points. Though certainly, perhaps in all complaints, as well as in the plans by which they ought to be removed, there are certain general and perhaps invariable circumstances attending them; yet attention to these alone, and a neglect of other occasional occurrences, have often been productive of the most fatal consequences. It is owing to this, that attention to the existing circumstances will often enable us to be successful practitioners, when the contrary will often involve us in embarrassments and perplexities from which we can only extricate ourselves with the greatest difficulty.

When the ulcers then appear in an apparently sound and vigorous person, they are usually easily remedied by simple means; but in debilitated and scrofulous habits the difficulty in their removal seems to have been proportioned to the existing derangement. In the first, cleanliness, simple dressings, with occasionally the internal use of bark and wine, have entirely removed them in a short time; but in the latter, more active measures were absolutely necessary. I have elsewhere suggested a plan for the removal of such affections; and in the present instance I find it invariably to answer my most sanguine expectations. What I allude to is, the internal use of cantharides, of the successful application of which I formerly inserted some remarks in this Journal. To them I

now now refer my readers; but in a distinct publication, the printing of which is about finished, I have entered still more fully into a consideration of this important subject.

The other most prevalent complaint is dropsy, of which various cases have occurred in practice. This disease indeed had, seemingly in every form, been gradually approaching from the very commencement of the month; and although there are various cases both of ascites and hydrothorax, yet the most generally prevailing form of the complaint is that of anasarca. The majority of the cases, in whatever form they appeared, were removable by small and repeated doses of supertartrite of potass, pills composed of calomel and squills, and tincture of cantharides. Some of them, however, in the form of hydrothorax, or in that of ascites, required that the fluid should be removed by surgical means; yet, even then, the medicines now mentioned were obliged to be continued after the operation for a considerable length of time.

In one case of ascites, after every internal medicine was employed without producing any remarkably beneficial effects, the operation was attempted; but to the mortification of the surgeon, who never before had performed an operation of the kind, no fluid followed on withdrawing the trocar. It was again introduced at a different part, but was still unsuccessful. The patient continued to labour under his accumulating miseries for about a week more, when he died. On dissection, the causes of the failure were evident, for the fluid had collected in cysts, to which the trocar could not reach. Besides this, the whole abdominal viscera seemed one mass of disease.

*Princes Street.*

